# Analysis of Foveal and Parafoveal Microvascular Density and Retinal Vessel Caliber Alteration in Inactive Graves' Ophthalmopathy

**DOI:** 10.1155/2020/7643737

**Published:** 2020-03-19

**Authors:** Cetin Akpolat, Muhammed M. Kurt, Merve Yılmaz, Fikriye Ordulu, Ferhat Evliyaoglu

**Affiliations:** ^1^Department of Ophthalmology, Sisli Hamidiye Etfal Training and Research Hospital, İstanbul, Turkey; ^2^Department of Ophthalmology, Samsun Gazi Community Hospital, Samsun, Turkey; ^3^Department of Endocrinology, Samsun Gazi Community Hospital, Samsun, Turkey; ^4^Department of Ophthalmology, Umraniye Training and Research Hospital, İstanbul, Turkey

## Abstract

**Purpose:**

We aimed to evaluate foveal and parafoveal density using optical coherence tomography angiography and the alteration on the retinal vessel diameter in patients with inactive Graves' ophthalmopathy compared to age-matched normal population. *Materials and Methods*. Patients with inactive Graves' ophthalmopathy (study group) and healthy individuals (control group) were enrolled in the cross sectionally designed study. The optical coherence tomography angiography parameters and retinal vessel diameter measurements were assessed between the study and control groups. Foveal and parafoveal microvascular density in the retina was measured using optical coherence tomography angiography. Retinal artery and vein diameter and artery/vein ratio were assessed for retinal vessel caliber changes.

**Results:**

Patients with inactive Graves' ophthalmopathy had higher values of intraocular pressure, proptosis, and axial length (*P*=0.001, *P*=0.001, *P*=0.001, *P*=0.001, *P*=0.001, *P*=0.001, *P*=0.001, *P*=0.001, *P*=0.001, *P*=0.001, *P*=0.001,

**Conclusion:**

Optical coherence tomography angiography could be a novel and promising noninvasive diagnostic technique in patients with inactive Graves' ophthalmopathy to detect foveal and parafoveal vessel density changes compared to healthy subjects. The decrease of retinal vessel diameter might be observed in patients with inactive graves ophthalmopathy.

## 1. Introduction

Graves' disease (GD), with an approximately 5% incidence, is a systemic autoimmune disease-causing various impairment of the thyroid gland, skin, and orbit [1]. Thyroid eye disease (TED) is the same as Graves' ophthalmopathy (GO) or thyroid-associated orbitopathy (TAO). Nearly half of patients with manifestations of deep orbital discomfort, tearing, photophobia, visual disturbances, eyelid retraction, exophthalmos, lagophthalmos, strabismus, diplopia, and optic neuropathy may develop TED [2]. These signs are caused by the overcompression of orbital tissues within the surrounding orbital bone cavity. Although the certain mechanism of TED is not clear, it is thought that the circulating antibodies produced against the antigens (such as the thyroid-stimulating hormone receptor (TSH-R), thyrotropin receptor, and insulin-like receptor) of the thyroid gland may also attack the orbit which also has the similar antigens like the thyroid gland [3]. This leads to activation of cytokine cascade, chemokines and orbital fibroblast proliferation and mucopolysaccharide infiltration, inflammatory causing cells, T/B cells, and immune complexes. All these cause inflammatory reaction in the extraocular muscles and connective tissue of the orbit, resulting in an increase in extraocular orbital volume and pressure [4]. GO has clinical courses of active and inactive inflammatory cycles, and the amount of autoantibodies correlates positively with the clinical severity of the disease [5]. The symptoms of GO diminish in most of the patients spontaneously. However, nearly 15% of the patients have significant signs, and 5% of the patients develop severe signs of thyroid orbitopathy [6].

Improvements in technology have provided evolutions in retinal imaging since the introduction of optical coherence tomography (OCT) in 1991 [7]. Being comparable to the histological microscopy of the retina makes OCT a unique imaging device. OCT is an essential imaging modality to guide and manage retinal diseases [8]. Due to OCT's structural imaging nature, it is inadequate to evaluate functional and dynamic changes in the retina. So, it does not provide information about the retinal vasculature and numerical blood flow assessment in the retina and optic nerve head (ONH) [9]. Therefore, fluorescein angiography (FA) and indocyanine green angiography (ICGA) still remain the standard imaging modalities to visualize blood vessels and the dynamic changes within the retinal vasculature. FA and ICGA have some limitations such as intravenous dye administration, time consuming (up to 20 minutes), absence of topographic 3-dimensional (3D) images, low image resolution, and difficulty in the quantification of findings [10]. The introduction of OCT angiography (OCT-A) has solved these issues and provides rapid, noninvasive, high-resolution 3D images, and reliable quantitative data from the retinal and choroidal vasculature and structures [11–13].

In the present study, we aimed to observe and assess the functional and dynamic changes in the retinal vasculature using OCT-A and retinal artery or vein diameter alteration in patients with inactive GO.

## 2. Materials and Methods

The present study was acted according to the tenets of the Helsinki Declaration, and the medical ethics committee approved the study. Each participant signed informed consent before the participation in the study.

### 2.1. Patients

Fifty-eight eyes of 29 patients with inactive GO and 60 eyes of 30 age-matched normal individuals were included in this cross-sectional study. Inactive GO was evaluated using the NOSPECS score [14]. The eyes of patients with inactive GO served as the study group, and the eyes of normal individuals comprised the control group. All participants had a detailed ophthalmic examination including best-corrected visual acuity (BCVA), biomicroscopic anterior segment and fundus examination, color fundus photography, intraocular pressure (IOP, mmHg), and OCT-A measurements. The BCVA was determined based on the Snellen chart and expressed in decimal. The eyes with high intraocular pressure (>21 mmHg), ocular inflammation, refractive error >6 diopters, diabetic retinopathy or any other choroidal/retinal disorders, cataract, and OCT-A images with a quality of <6 were not included in the study. Patients with clinical activity score (CAS) ≥ 3, pregnant or lactating subjects, and individuals who had eye surgery within 6 months were also excluded.

CAS, spherical equivalent in diopter (SE, D), proptosis level (PROP, mm), central corneal thickness (CCT, *μ*m, measured with TRK-2P which is a noncontact tonometer and pachymeter manufactured by Topcon), axial length (AXL, mm), Schirmer (mm), and tear breakup time in second (TBUT, sc) were the measurements performed before OCT-A measurements.

### 2.2. OCTA Measurement System

**Figure 1:**
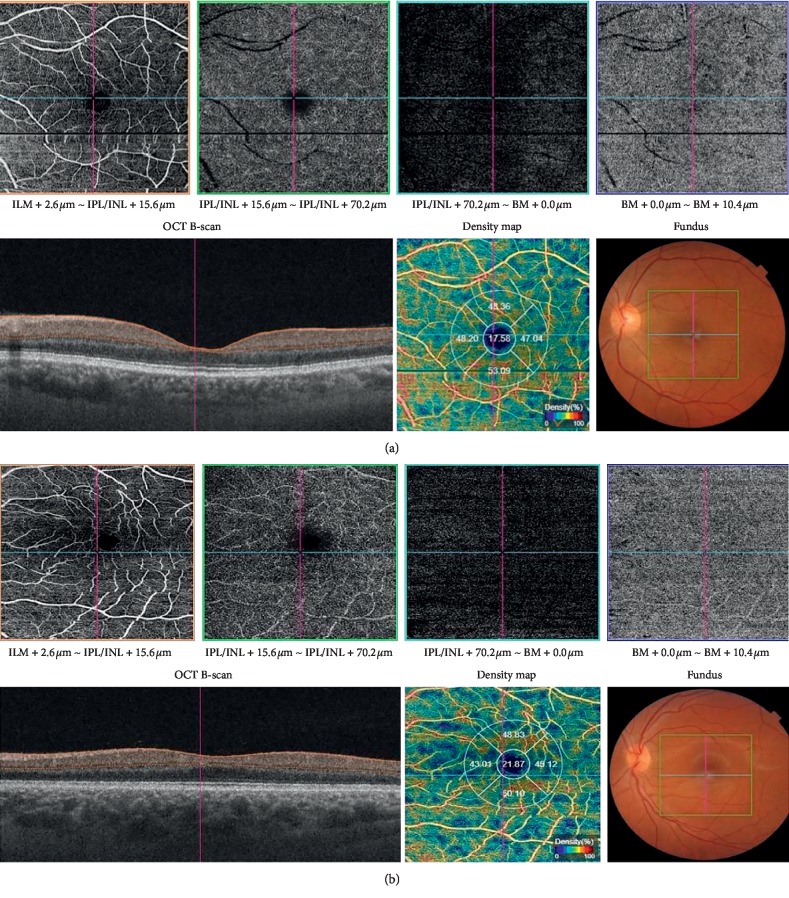
The measurements of foveal and parafoveal vessel density in a (a) patient with inactive GO and (b) control subject.

Triton model OCT-A (Topcon DRI OCT Triton swept-source OCT; Topcon, Tokyo, Japan) using the so-called OCT-A ratio analysis (OCTARA) algorithm was used to obtain the fundus angiography images. Triton has 1050 nm wavelength, 100.000 A-scans per second, and 8 *μ*m axial and 20 *μ*m transverse resolution. Sections measuring 3 × 3 mm [2] centered on the fovea were obtained using OCT-A. The measurements of retinal thickness and foveal and parafoveal vessel density were obtained (Figure 1). Central retinal thickness (CRT) was expressed as the mean thickness of the retina within the central of 1 mm diameter ring. The “fovea” was defined as the area encompassing the central fovea where there are no vessels. The “parafovea” was defined as an annulus centered on the fovea with inner and outer ring diameters of 1 mm and 3 mm, respectively. Vessel density is a measurement of the proportion of pixels occupied by flowing vasculature of all pixels including the region for analysis. Foveal (within 300 *μ*m) and parafoveal vessel density in superior, temporal, inferior, and nasal segments were measured using new three-dimensional software with artifact removal. The outlining of the foveal boundary is automated by the machine [15, 16].

### 2.3. Retinal Vasculature Diameter Measurement System

**Figure 2:**
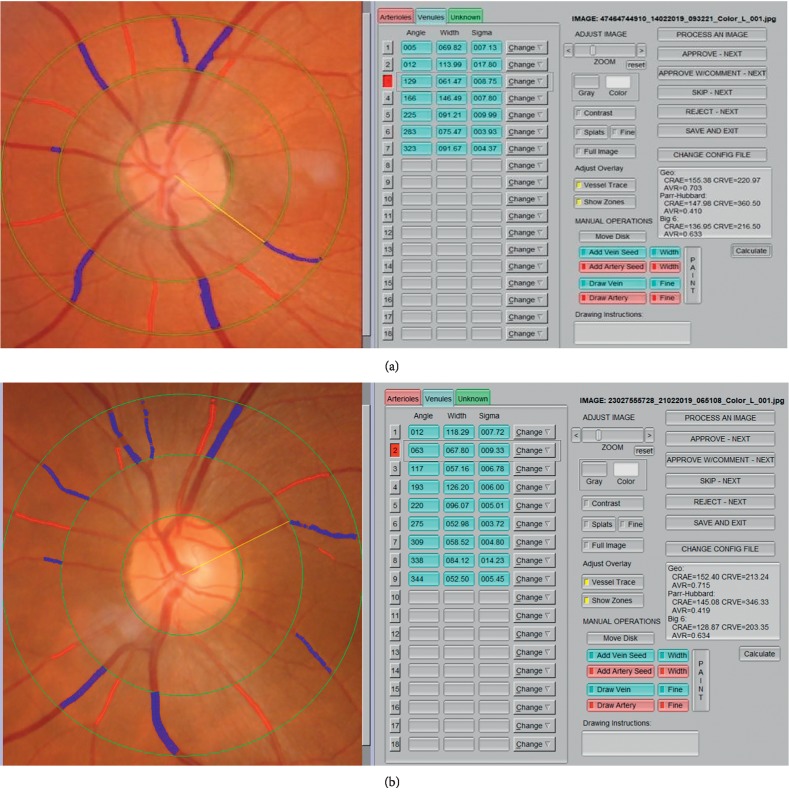
The measurements of CRAE and CRVE parameters in a (a) patient with inactive GO and (b) control subject.

The mean vessel caliber of the largest 6 retinal arteries was evaluated. The retinal vascular diameters were expressed as the central retinal artery equivalent (CRAE, *μ*) and central retinal vein equivalent (CRVE, *μ*). IVAN (University of Wisconsin, Madison, WI, USA) program was used to measure the retinal vasculature caliber. A masked researcher measured the retinal vasculature caliber. CRAE and CRVE parameters were measured with the aid of the formula created by Hubbard and revised by Knudtson (Figure 2) [17, 18].

Prior to study measurements, the rate of pulse, blood pressure, and respiration for the subjects was measured following a 5 min resting in the sitting position. Fundus examination and IOP measurements were repeated if the participants did not have similar values of blood pressure, pulse rate, and respiration rate compared to baseline.

### 2.4. Statistical Analysis

The data analyses were performed using SPSS for Windows version 21.0 software (IBM Corp., Armonk, NY, USA). Constant parameters were defined as the mean ± standard  deviation. Kolmogorov–Smirnov test was used for the suitability of the parameters to normal distribution. Differences in the data obtained from the groups and microvessel densities in sectors between the groups were assessed using Student's *t*-test. All statistical tests were considered significant when the *p* value was <0.05.

## 3. Results

The mean age of the study group was 40.66 ± 11.83 (22–48) years and of the age-matched control group was 42.60 ± 12.38 (27–52) years (*P*=0.322). Fifteen patients (51.72%) in the study group were male, and the left (14, 48.28%) were female. Twelve subjects (40.00%) were male, and 18 subjects (60.00%) were female in the control group. The mean time duration for Graves' disease was 2.22 ± 3.53 years (0.2–19 years). Twenty-six patients (89.65%) had medical treatment, and 3 patients (10.35%) had radioactive iodine treatment for Graves. Twenty-three patients (79.32%) had antithyroid treatment, 4 patients (13.79%) had thyroid treatment, and 2 patients (6.89%) had no treatment for the maintenance treatment of Graves. Fifteen patients (51.72%) were in hyperthyroidism, 6 patients (20.69%) were in hypothyroidism, and 8 patients (27.59%) were euthyroid.

The mean pre-OCT-A features of the patients in the study group and subjects in the control group are shown and compared in Table 1. IOP in the study group had a significant higher mean than in the control group (*P*=0.001). Compared with the control group, the mean proptosis value of the study group was significantly higher (*P*=0.002). Patients with inactive GO possessed a longer axial length than the normal-aged subjects (*P*=0.008).

The mean OCT-A measurements are shown in Table 2. While temporal and nasal parafoveal vessel density values were significantly higher in the study group than in the control group, foveal and other segments of the parafoveal area had similar vessel density values in the study and control groups.

The eyes of healthy age-matched subjects had significantly higher CRAE, CRVE, and AVR values than the eyes of the patients with inactive GO (*P* ≤ 0.001, *P*=0.033, and *P* ≤ 0.001, respectively) (Table 3).

We did not find any statistically significant relationships between the foveal vessel density, superior/temporal/inferior/nasal parafoveal vessel density, and BCVA (*r* = −0.058, *P*=0.535; *r* = 0.017, *P*=0.852; *r* = 0.093, *P*=0.318; *r* = −0.015, *P*=0.868; and *r* = 0.001, *P*=0.988, respectively) in the study group. We noted a highly strong negative correlation between the disease duration and BCVA (*r* = −0.914, *P* ≤ 0.001). We also observed strong positive correlations among the vessel densities of parafoveal segments in the study group (superior/temporal parafoveal: *r* = 0.421, *P* ≤ 0.001; superior/inferior parafoveal: *r* = 0.347, *P* ≤ 0.001; superior/nasal parafoveal: *r* = 0.458, *P* ≤ 0.001; inferior/temporal parafoveal: *r* = 0.527, *P* ≤ 0.001; temporal/nasal parafoveal: *r* = 0.723, *P* ≤ 0.001; and inferior/nasal parafoveal: *r* = 0.535, *P* ≤ 0.001). Statistically significant correlations were not observed between any other parameters.

## 4. Discussion

GO is mostly clinically diagnosed and supported by laboratory studies accompanied by thyroidal gland impairment and presence of antithyroid antibodies. CT and MRI have been used in the imaging of orbit to assess whether GO is active or severe and provide a pilot way for the progress of the disease. Intensive attention has been demonstrated on the novel methods in the assessment of optic nerve compression, an unusual and undesirable complication of GO. Color Doppler ultrasound-guided superior ophthalmic vein flow measurement and oculodynamometry-determined venous outflow measurements are among objective old techniques to assess the degree of the impairment [19, 20]. However, these modalities may have some restrictions such as radiation, invasiveness, and difficulty in practice and repetition. In contrast to all these studies, OCT-A provides a high and 3D resolution. OCT-A also offers us to compare the anatomy and the circulation of the fundus. So, we can use OCT-A in the evaluation of compressive orbital disease.

OCT-A is a new technique to measure retinal blood flow by differentiating the mobile (blood flow) and stable structures. OCT-A works with time-related differences in erythrocytes reflective to render flow maps of retinal circulation with segmentation capabilities [21]. OCT-A benefits from the red blood cells as contrast agents instead of fluorescein angiography to visualize the retinal blood flow. Additionally, cross-sectional images provide multidepth assessment of retinal microvessels.

OCT-A provides qualitative data and quantitative measurement of the microcirculation and the vessel density of the fovea, parafovea, peripapillary region and ONH in diabetic retinopathy, central serous chorioretinopathy, age-related macular degeneration, glaucoma, and other disorders [22]. However, there is no relevant study investigating the involvement of vessel densities in the macular retina in patients with inactive GO. To our best knowledge, this is among the first applications of swept-source OCT-A technology in inactive GO. In the present study, using OCT-A and fundus photography, foveal and parafoveal vessel density and retinal vessel diameter of the patients with inactive GO were evaluated to be compared with age-matched normal subjects.

Hyperthyroidism is associated with elevated systemic pressure, increased cardiac output, higher pulse pressure, tachycardia, and decreased systemic vascular resistance and diastolic blood pressure, and as a result, increased cardiac output may enhance orbital blood flow [23]. Several studies have concluded an increase in retinal blood flow and retinal artery resistance and a reduction in the peak systolic velocity (PSV) and end diastolic velocity (EDV) of the central retinal artery in GO patients [24–26]. However, Alimgil et al. [27] revealed that ocular blood flow was significantly decreased in GO patients and claimed that this was the consequence of increased venous pressure and choroidal vessel resistance due to elevated intraorbital pressure. Technical differences and variation in patient status, such as inactive or active TAO state or moderate-to- severe degree of GO, might at least partially explain the discrepancy [28].

In our literature review, we have found a variety of studies which are focusing on orbital blood flow in patients with TAO. It is known that there is some kind of inflammatory changes going in orbital spaces which finally results in compression of the orbit. Compression of the orbit causes some kind of pressure on the venous system which can lead to retro blood flow. Orbital inflammation increases orbital arterial blood flow velocities in most GO patients [29], and inflammatory changes cause congestion around and that results in an elevated episcleral venous pressure, and these changes make an obstruction effect on the central retinal vein [30]. Hemodynamic changes in TAO, such as decreased flow velocity and stasis in the superior ophthalmic vein, are well documented with Doppler ultrasonography in a previous study [31]. In the literature, there are a variety of studies reported that there is reverse venous flow in patients with TAO [32, 33].

Retinal vessel diameter could be affected by external and systemic factors [34]. Central retinal artery blood flow has also been investigated in patients with TAO, and different results have been reported [35, 36]. These studies were focusing on blood flow in orbital vessels, which is also related with retinal vessel diameter. In our study, we aimed to measure directly retinal vessel diameter which can be accepted as a good indicator of blood flow. In a study, Yang et al. [37] focused on retinal vessel diameter, and consequently, they reported that severe inactive GO may have some effects on retinal veins, but may not affect retinal arterioles, which means that a reduction in retinal venous caliber might point severe GO. Some studies also concluded that severe TAO might cause a reduction in the blood flow of the retinal vein, which may be related with the narrower diameter of the retinal vein [38, 39]. Although we do not have enough knowledge regarding the reason for the reduction of retinal vein caliber in eyes with severe inactive GO, some studies in the literature have attributed this narrowing of the retinal vein diameter to the atrophy of the retina and the reduction of retinal blood flow [40, 41]. Similarly, we observed lower retinal vasculature caliber values in subjects with inactive GO when compared to age-matched normal subjects. We can explain the reduction of retinal vein diameter as a result of increased venous pressure in Graves' disease following extraocular muscle and orbital volume increase caused by inflammation. Although there is an increase in retinal artery blood flow, the reduction in retinal artery diameter might be due to the increase in retinal artery vascular resistance which could be the result of overperfusion caused by increased cardiac output [42].

Theoretically, several possible factors may lead to changes in retinal vessel density in patients with inactive GO, including systemic hypertension, hyperthyroidism, ocular hypertension, and/or orbital inflammation [43]. There were limited studies in the literature regarding the vessel density in inactive GO. Tehrani et al. [44] conducted a study to compare macular and peripapillary vasculatures in active thyroid eye disease (TED), not active not compressive (NANC) TED, and control eyes. In contrast to us, they have concluded that whole macular and parafoveal superficial vessel densities were significantly lower in the active TED compared with the control group. Ye L et al. [45] used OCT-A imaging to characterize the retinal microvasculature in active TAO patients. Their results demonstrated that the macular and microvessel density was altered in both superficial and deep retinal layers, and vessel density may influence visual acuity. We noted alterations in foveal and parafoveal vessel density in patients with inactive GO, but we did not find any association between vessel density and visual acuity. In another study, Lewis et al. [46] reported increased vessel density in patients with active Graves who need orbital decompression surgery. They measured orbital vessel density with OCT-A. They reported a significant decrease in vessel distension in the postsurgical period.

In our study, we have found that retinal vessel diameter decreased and macular vessel density increased in inactive GO. These changes could be affected by circulation systems (retinal and choroidal) supplying the posterior segment of the eye. The microcirculation of the superficial retinal layer is only provided by retinal circulation which is regulated by local factors produced in endothelial cells. The deeper retinal layer and macular area are nourished by choroidal circulation, which is mostly regulated by perfusion pressure and sympathetic innervation due to less autoregulation [47].

Lack of a comparative group of active patients with disease stage and relatively small sample size are of the limitations of the present study. Regarding these limitations, further studies with a large population including the comparison of active and inactive GO and comparison by disease stage can be conducted.

In conclusion, the current study revealed the comparison of retinal vessel density in the foveal and parafoveal area and retinal vessel diameter in patients with inactive GO and age-matched normal subjects. OCT-A might be a valuable noninvasive method of characterizing retinal microvascular network changes and quantifying the foveal and parafoveal vessel density. This provides us to assess the retinal blood flow of eyes with GO, and thus, we can predict the eyes with high risk of optic nerve compression. So, it might be a useful tool for enabling further understanding of GO pathogenesis, observing the GO progression and optic neuropathy developing. OCT-A also might be a model to evaluate the efficiency of medical and/or surgical therapies. An important advantage of OCT-A is that it is a much more objective imaging tool, almost independent of the examiner's skill. Retinal vessel diameter alterations might also be observed in patients with GO, which can be accepted as a good indicator of ocular blood flow. We also observed a negative strong relation between disease duration and BCVA. The clinical relevance of the present study is that the outcome of the present study might give us an ophthalmologic clue about the time of treatment intervention in patients with GO.

## Figures and Tables

**Table 1:** Comparison of mean pre-OCT-A features of the eyes of patients in the study group and the eyes of the subjects in the control group (study group, *n* = 58; control group, *n* = 60).

Features	Study group	Control group	*P* ^*∗∗*^
BCVA (decimal)	0.99 *±* 0.067	1.00 *±* 0.00	0.232
CAS	0.10 ± 0.41	0.00 *±* 0.00	0.051
IOP (mmHg)	16.88 ± 2.28	15.02 ± 2.56	0.001^*∗*^
SE (D)	−0.25 ± 0.90	−0.09 ± 3.75	0.200
PROP (mm)	12.31 ± 1.70	11.58 ± 0.49	0.002^*∗*^
CCT (*μ*m)	549.64 ± 29.31	546.35 ± 33.96	0.575
AXL (mm)	23.02 ± 1.03	22.63 ± 0.42	0.008^*∗*^
Schirmer (mm)	15.16 ± 2.59	14.65 ± 1.77	0.217
BUT (sc)	14.91 ± 2.96	14.68 ± 2.09	0.626

^*∗∗*^Statistical analysis was calculated by independent samples *t*-test, ^*∗*^statistically significant, BCVA: best-corrected visual acuity, CAS: clinical activation score, IOP: intraocular pressure, SE: spherical equivalent, PROP: proptosis level, CCT: central corneal thickness, AXL: axial length, BUT: breakup time, and sc: second.

**Table 2:** Comparison of the mean OCT-A measurements of the eyes of patients in the study group and the eyes of the subjects in the control group (study group, *n* = 58; control group, *n* = 60).

Density (%)	Study group	Control group	*P* ^*∗∗*^
CRT	226.10 ± 19.78	231.55 ± 28.38	0.230
Foveal	21.73 *±* 5.97	21.65 *±* 4.84	0.268
Parafoveal_S	50.37 ± 3.31	49.45 *±* 2.84	0.107
Parafoveal_T	48.93 ± 3.21	47.62 ± 2.29	0.017^*∗*^
Parafoveal_I	50.73 ± 3.07	49.60 ± 3.22	0.055
Parafoveal_N	47.55 ± 3.01	46.46 ± 2.57	0.035^*∗*^

^*∗∗*^Statistical analysis was calculated by independent samples *t*-test, ^*∗*^statistically significant, CRT: central retinal thickness, Parafoveal_S: superior parafoveal vessel density, Parafoveal_T: temporal parafoveal vessel density, Parafoveal_I: inferior parafoveal vessel density, and Parafoveal_N: nasal parafoveal vessel density.

**Table 3:** Comparison of mean retinal vessel diameter measurements of the patients in the study group and the subjects in the control group (study group, *n* = 30; control group, *n* = 30).

Measurements (*μ*)	Study group	Control group	*P* ^*∗∗*^
CRAE	149.15 *±* 15.87	172.61 *±* 18.61	≤0.001^*∗*^
CRVE	211.08 ± 24.96	220.28 *±* 21.41	0.033^*∗*^
AVR	0.71 ± 0.08	0.79 ± 0.08	≤0.001^*∗*^

^*∗∗*^Statistical analysis was calculated by independent samples *t*-test, ^*∗*^statistically significant, CRAE: central retinal artery equivalent, CRVE: central retinal vein equivalent, and AVR: artery-to-vein ratio.

## Data Availability

The data used to support the findings of this study are available from the corresponding author upon request.
